# Fabrication and Characterization of Electrospun Membranes Based on “Poly(ε-caprolactone)”, “Poly(3-hydroxybutyrate)” and Their Blend for Tunable Drug Delivery of Curcumin

**DOI:** 10.3390/polym12102239

**Published:** 2020-09-28

**Authors:** Giuliana Gorrasi, Raffaele Longo, Gianluca Viscusi

**Affiliations:** Department of Industrial Engineering, University of Salerno, Via Giovanni Paolo II, 132, 84084 Fisciano (SA), Italy; rlongo@unisa.it (R.L.); gviscusi@unisa.it (G.V.)

**Keywords:** poly(ε-caprolactone), poly(3-hydroxybutyrate), electrospinning, curcumin, release

## Abstract

Membranes based on poly(ε-caprolactone)/poly(3-hydroxybutyrate) blends (PCL/PHB at 50 wt%) were obtained by electrospinning and curcumin encapsulated at 1 wt% as active agent, as drug delivery systems for biomedical applications. PCL and PHB were also separately electrospinned and loaded with 1 wt% of curcumin. The processing parameters of PHB were drastically different from PCL and the blend PCL/PHB; in fact, the temperature used was 40 °C, and the distance injector–collector was 28 cm. Different conditions were used for PCL: lower temperature (i.e., 25 °C) and shorter distance injector–collector (i.e., 18 cm). The blend was processed in the same conditions of PCL. The fibers obtained with PHB showed diameters in the order of magnitude of micron (i.e., ≈ 3.45 µm), while the PCL mats is composed of fiber of nanometric dimensions (i.e., ≈ 340 nm). PCL/PHB blend allowed to obtain nanometric fibers (i.e., ≈520 nm). Same trend of results was obtained for the fibers’ porosity. The morphology, thermal, mechanical and barrier properties (sorption and diffusion) through water vapor were evaluated on all the electrospun fibers, as well as the release behavior of curcumin, and correlated to the processing parameter and the fibers’ morphologies.

## 1. Introduction

Electrospinning process represents an excellent and versatile technique for the fabrication of micro- and nanofibers using almost any type of polymer, either synthetic or natural [1,2,3,4,5,6]. The technological applications of electrospun fibers are many and diversified, i.e., filtration, protective clothing, wound healing and biomedical applications [1,7,8,9,10,11,12,13,14]. Regarding the biomedical applications, the use of electrospun fibers has been demonstrated to be very promising in particular for scaffolds in tissue engineering and drug delivery [15,16,17,18,19,20,21]. The main advantages of using electrospun fibers as drug delivery systems are high drug loading capacity, high surface area, good mechanical properties, porosity and capability to delivery simultaneously different therapeutic agents [22,23,24,25,26,27,28,29,30,31,32]. Delivery systems based on electrospun membranes, in which drugs are encapsulated and released at controlled rates for long times (i.e., from days to months) need deep investigation, in terms of materials employed and relative amount, drug loading and processing parameters, in order to overcome the limitations of traditional administration routes that are generally very fast release systems (from minutes to hours). Furthermore, the drug release rates can be tailored to the needs of a specific application: (i) controlled release systems provide protection of drugs that are otherwise rapidly destroyed in the patient’s body; (ii) some diseases are treated most effectively by maintaining a relatively constant drug concentration within a certain therapeutic range, which may require a constant rate of drug delivery; (iii) encapsulation of pharmacologically active molecules into fibers enhances the solubility of poorly water-soluble drugs. The use of biodegradable materials for these purposes, pure or in blend, allows to elongate the release times, even to years. Therefore, drug release can be due either to erosion or to diffusion. The release mechanism will in turn affect the drug pharmacokinetics, which is critical for determining drug performance. Drug release from biodegradable polymers or blends in vivo is generally governed by a combination of both mechanisms and therefore depends on the relative rates of erosion and diffusion [33,34,35]. Natural products are the sources of widely used drugs. Among them, one of the most promising for several diseases and also abundant is curcumin. Curcumin, known as (bis-1,7-[4-hydroxy-3- methoxyphenyl]-hepta-1,6-dione), is derived from *curcuma longa* [36,37] and the yellow pigment known as turmeric [38]. It has great potential for the development of a therapeutic, natural turmeric being classified as a GRAS material [39]. The use of curcumin has been reported for many medical applications, due to its outstanding properties, such as anti-inflammatory [40,41], antioxidantv [42], antifungal [43] and antibacterial [44]. Other studies report the use of curcumin for specific diseases and/or functions: as an anticancer agent [45,46], a therapeutic for Alzheimer’s disease [47], a treatment for hangovers [48], erectile dysfunction [49], baldness [50,51], fertility boosting [52] and as a contraceptive [53]. Poly(ε-caprolactone) (PCL) is one of the most used biodegradable polymers used in drug delivery but has long degradation times (2–4 years) under ambient conditions [54]. An interesting strategy to fabricate PCL based materials for biomedical applications is to blend such polymer with biopolymers with fast degradation rate. Polyhydroxyalkanoates are a family of polymers produced by various microorganisms and applied alone or in combination with other materials in medical applications, such as release of drugs, tissue repair and patches [55]. Poly(3-hydroxybutyrate) (PHB) is a member of the polyhydroxyalkanoates family that has a higher degradation rate than polymers of poly-alpha-hydroxy-acids, such as poly-lactic acid and PCL [56]. It has been reported that PCL/PHB blends have been employed in artificial tissue textures for regenerating bone, tendon and cartilage [57,58]. The present paper reports the preparation and characterization of electrospun membranes based on PCL and PHB and their blend at 50 wt%, loaded with curcumin (1% wt) as biodegradable drug delivery systems. The morphological analysis of the membranes was studied and correlated to the thermal, mechanical, barrier properties to water vapor and to the hydrophilicity degree. The controlled release of curcumin was analyzed as function of time and correlated to the composition and morphology of the electrospun membranes.

## 2. Materials and Methods

### 2.1. Materials

Poly(ε-caprolactone) (PCL) in pellet form (Mw = 80,000 Da; CAS: 24980-41-4) and Poly[(R)-3-hydroxybutyric acid] (CAS: 29435-48-1) were purchased from Sigma Aldrich (Milan, Italy). Tetrahydrofuran (THF pure, CAS: 109-99-9), Ethanol (EtOH purity >96%-CAS: 64-17-5) and Phosfate Buffer Solution (PBS) (pH = 7 ± 0.02, CAS:7558-79-4) were purchased from Carlo Erba Reagents (Cornaredo-Milano). N,N-Dimethylformamide (DMF; CAS: 68-12-2) and Chloroform (CHCl3; CAS: 67-66-3) were purchased from Sigma Aldrich. Curcumin from *Curcuma Longa* (Curc) (Mw = 368.38 g/mol; CAS: 458-37-7) was purchased in powder form from Sigma-Aldrich.

### 2.2. Preparation of Curcumin-Loaded Membranes Using Electrospinning

The electrospun fibrous membranes were prepared by dissolving PCL in a solvent mixture of THF/DMF (50:50 *v*/*v*) at 12% *w*/*w* and PHB in a solvent mixture of CHCl_3_/DMF (9:1 *v*/*v*) at 6% *w*/*w*. The PCL/PHB blend electrospun membrane was prepared by dissolving both polymers (1:1 *w*/*w*) in a solvent mixture CHCl_3_/DMF (9:1 *v*/*v*) at 9% *w*/*w*. Curcumin was added to the polymeric solutions at the drug to polymer ratio of 0.1:9.9 (*w*/*w*) and mixed for 4 h at 40 °C, using a temperature controlled stirring plate (300 rpm) to obtain a homogenous solution. For convenience, the electrospun membranes obtained were hereafter labelled as PCL + Curc, PHB + Curc and PCL/PHB/Curc, respectively. Prior to electrospinning, the polymeric solutions were characterized by measuring the viscosity and conductivity; the results are presented in Table 1. The set of electrospinning conditions, optimized to produce fibrous mats without bead formation, is even reported in Table 1.

### 2.3. Methods

The viscosity of polymeric solutions was evaluated using a Thermo Haake VT550 rotational viscometer (Waltham, MA, USA). Each test was performed in triplicate.

The conductivity of the polymeric solutions has been measured on a Mettler Toledo Conductivity Sensor LE703 model (Columbus, OH, USA). The measurements were taken at room temperature for freshly prepared solutions.

Climate controlled electrospinning apparatus (EC-CLI, IME Technologies, Geldrop, The Netherlands) was used to produce fibrous membranes. A vertical setup was chosen to carry out the experiments. The diameter of the needle was 0.8 mm, and for all the experiments, a copper collector was used to recover the electrospun fibers.

Scanning electron microscopy (SEM) was carried out using a Quanta 200 F microscope in high-vacuum mode (Thermo Fisher Scientific). Before the analysis, electrospun membranes were covered with a thin film of gold using an Agar Automatic Sputter Coater (Mod. B7341, Stansted, UK) at 40 mA for 120 s prior the analysis.

Porosity Measurements of the membrane were obtained processing the SEM images with Matlab Software. The method used, as reported in the literature [59], is based on a conversion of each SEM image into a binary image. The fibers are converted in white pixels, whereas the black regions among the fibers are the pores of the mat. Then, thanks to *regionprops* function, it is possible to know the dimension of each pore, to obtain the pore size distribution.

Thermogravimetric analyses (TGA) were carried out in an air atmosphere with a Mettler Toledo TC-10 thermobalance from 25 °C to 600 °C at a heating rate of 10 °C/min.

Differential scanning calorimetry (DSC) analyses were carried out in N_2_ atmosphere with a Mettler Toledo DSC 822e from −60 °C to 300 °C at a heating rate of 10 °C/min.

Mechanical properties were evaluated, in tensile mode, at room temperature using a dynamometric apparatus INSTRON 4301 (Norwood, MA, USA). Experiments were conducted at room temperature with the deformation rate of 10 mm/min. Elastic modulus was evaluated in the deformation range of 0.2%. Data were averaged on five samples.

Release kinetics of curcumin were followed using a Spectrometer UV-2401 PC (Shimadzu, Japan). The tests were performed using rectangular samples (weight ≅ 100 mg) with an exposed area of about 3 cm^2^. Release kinetic was performed in artificial biologic fluid (PBS/EtOH 70:30 *v*/*v*) at 25 °C placing each sample in 25 mL of release medium. The solution was stirred at 100 rpm in an orbital shaker (VDRL MOD. 711+ Asal S.r.l., Milan, Italy). The release medium was withdrawn at fixed time intervals and replenished with fresh medium. The considered band was 431 nm. The thicknesses of the tested membrane are 0.20, 0.25 and 0.30 mm for PCL + Curc, PCL/PHB/Curc and PHB + Curc, respectively.

Barrier properties of water vapor were evaluated through a DVS automated multi-vapor gravimetric sorption analyzer, using dry nitrogen as a carrier gas. The temperature was fixed to 30 °C. Samples were exposed to increasing water vapor pressures obtaining different water activities a_w_ = P/P_0_ (from a_w_ = 0.1 to a_w_ = 0.6) where P is the partial pressure into the gravimetric chamber, and P_0_ is the saturation water pressure at the experimental temperature. The adsorbed water mass was measured by a microbalance and recorded as a function of time. From the sorption kinetics it was possible to derive the diffusion coefficient at each activity or partial pressure. Data averaged on three samples.

Water contact angle measurements were performed using a high-resolution camera. Droplets of distillate water (100 μL) were dispensed onto the fibrous mat. The contact angle was determined using Drop Analysis software. Five tests were carried out for each sample.

## 3. Results and Discussion

**Figure 1:**
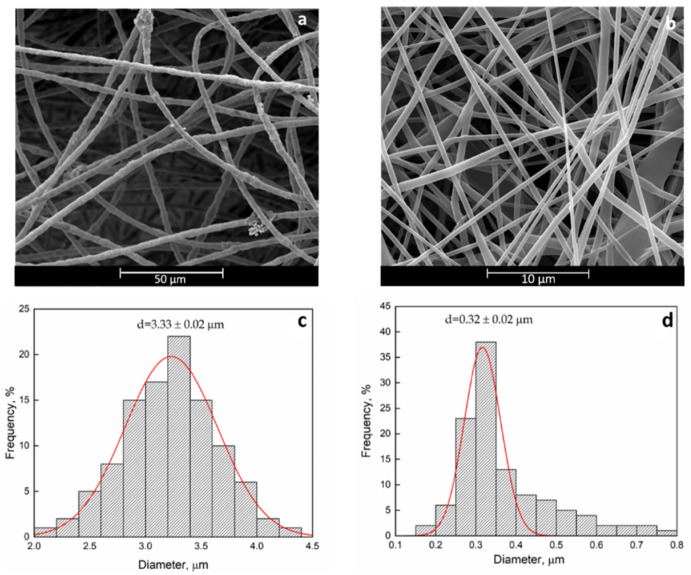
SEM and fiber diameter distribution of pristine Poly(3-hydroxybutyrate) (PHB) (**a**,**c**) and pristine poly(ε-caprolactone) (PCL) (**b**,**d**).

SEM analyses were performed in order to evaluate the effect of the processing parameters on the fibers’ morphology. The photographs of the pristine PCL and PHB electrospun membranes and the fiber diameter distributions are reported in Figure 1.

**Figure 2:**
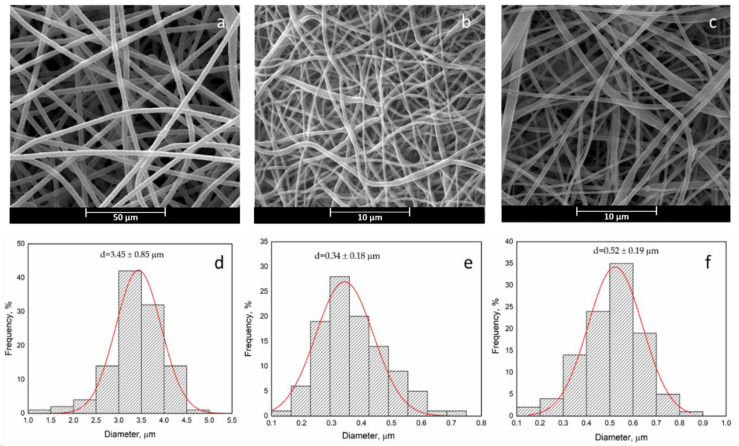
SEM image and fiber diameter distribution: PHB + Curc (**a**,**d**), PCL + Curc (**b**,**e**) and PCL/PHB/Curc (**c**,**f**).

Besides, Figure 2 reports the SEM graphs of curcumin loaded PCL and PHB membranes as well as the PCL/PHB blend membrane.

**Figure 3:**
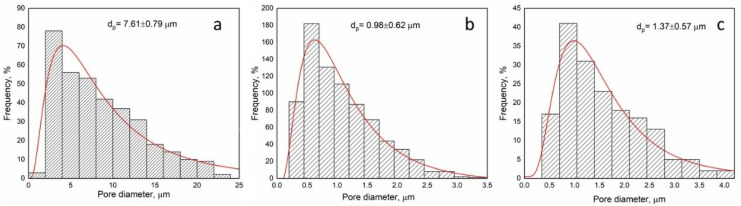
Pore Diameter Distribution of PHB + Curc membrane (**a**); PCL + Curc membrane (**b**); (**c**) PCL/PHB/Curc membrane.

The fiber diameter distributions are unimodal in all cases. Comparing the fiber diameter distribution, the morphology of pristine PCL and PHB is quite similar to the PCL/Curc and PHB/Curc, with no sensitive variations in the mean fiber diameter. It follows that the loading of curcumin did not affect the morphology of the electrospun membranes. The PCL + Curc membrane diameter (≈340 nm) is sensitively smaller than the PHB + Curc one (≈3.45 µm). This difference could be related to the lower viscosity and the higher conductivity of PCL solution compared to PHB one which enhanced the extensional stretching of the fibers. Moreover, the effect of molecular weight of the polymer is known to play an important role in producing thin fibers [60]. In fact, the molecular weight of PCL (80 kDa) is noticeably lower than PHB one (1 MDa). Moreover, since the molecular weight of the PHB led to a more viscous solution than PCL one, a higher resistance of the polymeric PHB jet to the extensional flow determines the achievement of fibers with higher mean fiber diameter respect to PCL membranes. The dimensions of the blend (≈520 nm), very close to the PCL’s mean diameter, are probably much more affected by the PCL properties. By the SEM images, the distribution of the pore dimensions in the fibers mat has been investigated. This parameter is relevant since electrospun fibers find wide application in the biomedical field (i.e., tissue engineering for the similarity to the human extracellular matrix [61,62]). The pore distributions are reported in Figure 3.

**Figure 4:**
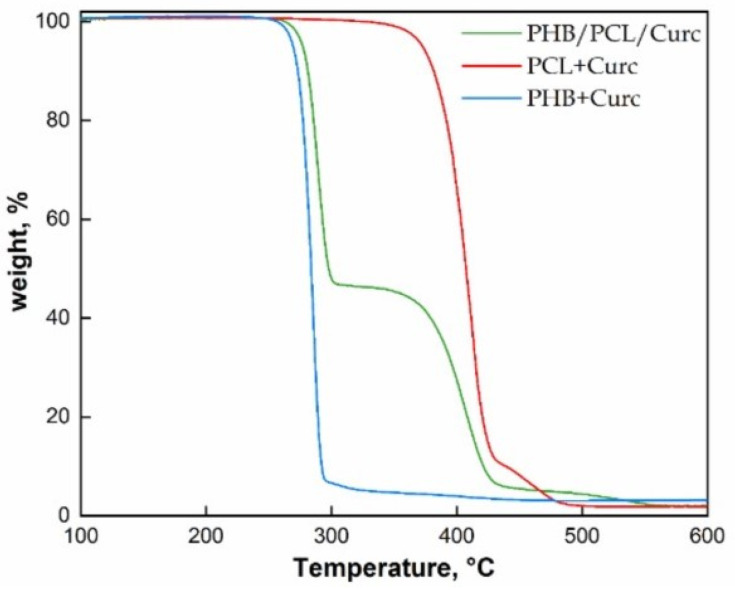
Thermogravimetric analyses (TGA) of electrospun PCL, PHB and PCL/PHB loaded with curcumin.

As for fibers’ dimensions, the porosity results are the highest for PHB membranes (≅7.61 µm), intermediate for the blend (≅1.37 µm) and the lowest for PCL (≅0.98 µm). It is worth to underline that the processing parameters of the blend are the same of PCL: 25 °C, compared to 40 °C of pure PHB, and shorter distance injector–collector 18 cm, compared to 28 cm for pure PHB. The processability of PHB, even at 50 wt% in the blend, results significantly improved in terms of experimental conditions used and allows to obtain fibers significantly thinner than the ones obtained using still PHB. Figure 4 reports the TGA evaluated on fibers of pure polymers loaded with curcumin and their blend.

The thermal degradation of PHB proceeds by a one-step process with a maximum decomposition temperature of ≅284 °C. Such loss in weight is mainly associated with the ester cleavage of PHB component by β -elimination reaction [63]. The degradation of PCL occurs into two steps of weight loss. The first one, centered at ≅407 °C, is due to a statistical rupture of the polyester chains via ester pyrolysis reaction with production of 5-hexenoic acid, H_2_O and CO_2_; the second one, centered at ≅454 °C leads to the formation of ε-caprolactone (cyclic monomer) as result of an unzipping depolymerization process [64]. The blend reveals a slight retard in the degradation of PHB (≅6 °C), due to the better thermal stability of PCL in blend, and a reduction of the degradation temperature of PCL (≅16 °C). This last effect could be due to the catalytic effect of the degradation products generated by the ester cleavage of PHB due to the β-elimination reaction [65].

**Figure 5:**
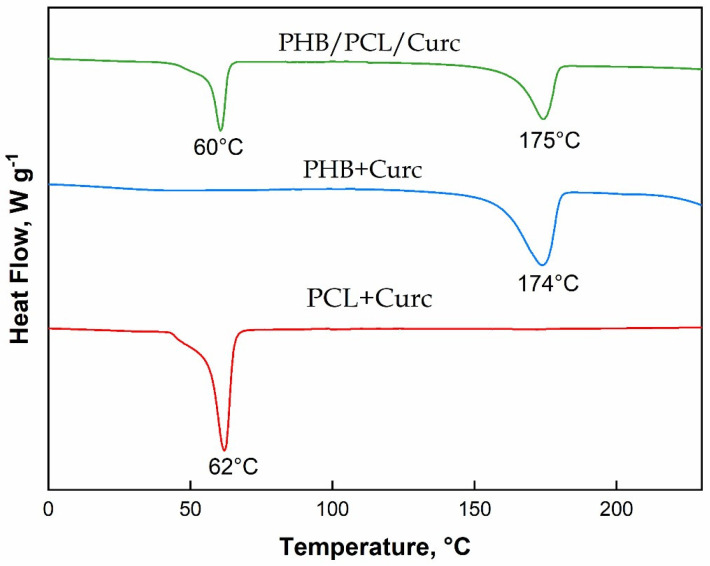
Differential scanning calorimetry (DSC) of electrospun PCL, PHB and PCL/PHB loaded with Curcumin.

Figure 5 reports the DSC evaluated on fibers of pure polymers and their blend. The crystallinity of the samples, X_c_ (%), were calculated from the melting enthalpy of the endothermic peaks from thermograms of Figure 4. It was evaluated according to Equation (1):

X_c_ (%) = ΔH_m_/ϕΔH_m0_ × 100 (1)

where ΔH_m_ is the melting enthalpy of PHB or PCL during the heating cycle, and ΔH_m0_ is the fusion enthalpy of 100% crystalline PHB that is 146.6 J/g [66] and of 100% PCL, that is 136.1 J/g [67]; ϕ is the weight fraction of PHB and PCL in the blend.

The melting temperatures of both polymers in blend are not significantly influenced by the presence of the second polymer. The degree of crystallinity, evaluated accordingly to Equation (1), is 40% for pure PCL and 56% for pure PHB and 35% and 52% for PCL and PHB, respectively, in the blend. This is an expected result because each polymer tends to hinder the crystallization of the other one, being both immiscible, as evidenced by the two distinct melting points in the thermogram of the blend.

**Figure 6:**
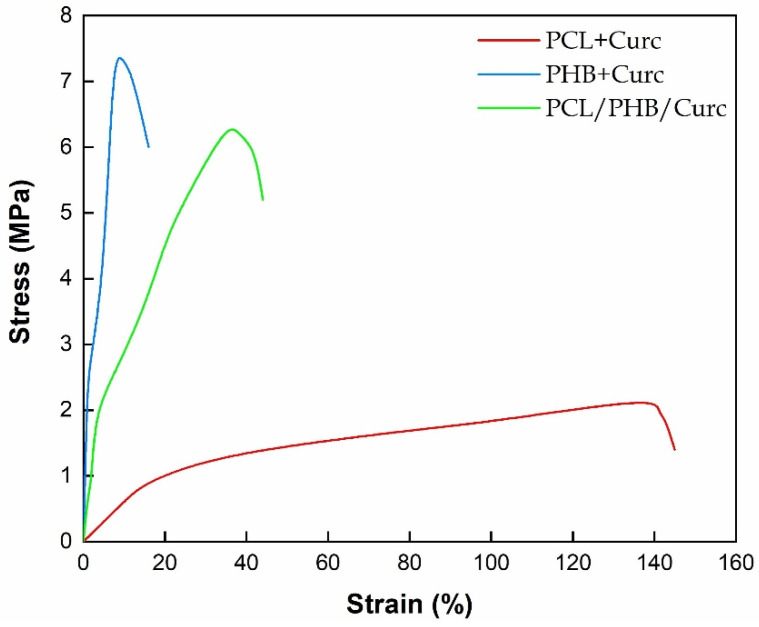
Stress–strain curves of electrospun membranes.

The mechanical properties of curcumin loaded electrospun fibrous membranes were evaluated from stress–strain curves (Figure 6).

**Figure 7:**
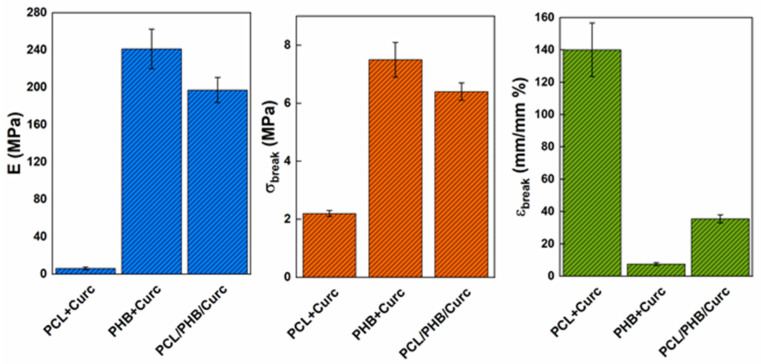
Elastic modulus, E (MPa), stress at break point, σ_b_ (MPa), and elongation at break point, ε_b_ (mm/mm%), for the electrospun membranes loaded with curcumin.

Figure 7 reports the elastic modulus, E (MPa), and stress at break point, σ_b_ (MPa) and elongation at break point ε_b_ (mm/mm %), extracted from Figure 6. PHB fibrous membrane shows good tensile strength (≅7 MPa), high elastic modulus (≅240 MPa) and low elongation at break (≅8%) compared to PCL + Curc values (E ≅ 6 MPa; σ_break_ ≅ 2 MPa; ε_break_ ≅ 140%). Moreover, the mechanical parameters of the blend PCL/PHB/Curc are intermediate between the PCL + Curc and PHB + Curc ones. PCL slightly reduced the mechanical performances of PHB membrane, in terms of elastic modulus and tensile strength, but increases the elongation at break. However, the barely decrease of these parameters does not compromise the mechanical properties of electrospun clend membrane.

**Figure 8:**
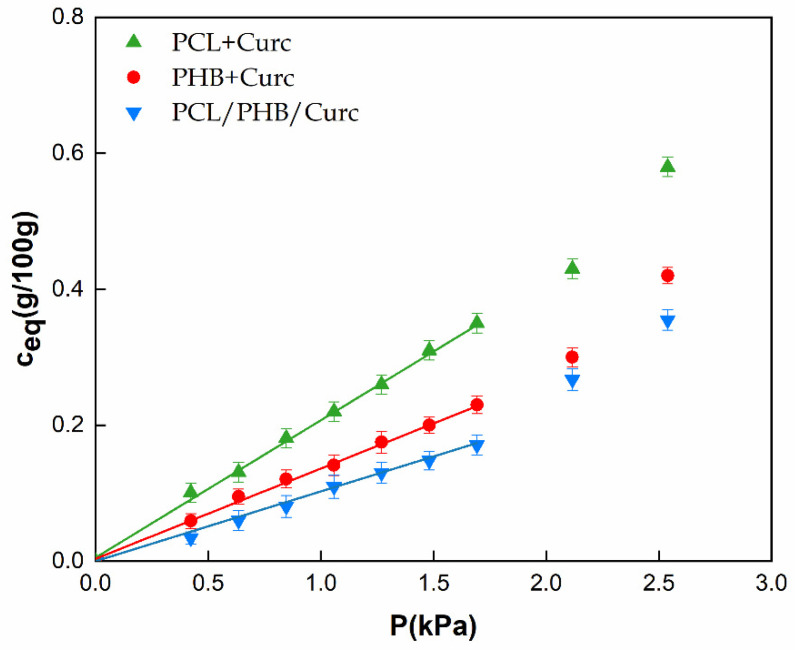
Sorption isotherms for electrospun membranes.

Barrier properties, sorption (S), diffusion (D) and permeability (S*D) to water vapor were evaluated on single electrospun polymers and their blends. In the case of biodegradable polyesters, for the presence of hydrophilic groups, the sorption of water can induce modification in the polymers’ arrangement into the manufactures (i.e., swelling, plasticization, hydrolysis) [68]. Being the curcumin a molecule with very low affinity to water, we can assume its contribution to the transport properties (S and D) to water is negligible. Figure 8 reports the equilibrium concentration of water vapor, C_eq_ (g/100 g)), as function of water partial pressure, P (kPa) for all the samples. All materials show an ideal behavior in the pressure range 0 ÷ 1.8 kPa. The sorption coefficient, S, was evaluated for all the samples using Henry’s law (Equation (2)) in this considered pressure range.

C_eq_ = S × P (2)

For pressures higher than 1.8 kPa, a deviation from the linearity for all the samples is evident, which follows a Flory–Huggins mode of sorption [69]. According to this model, the first sorbed molecule locally loosens the polymer structure allowing an easier entry for the following penetrant molecules. Such types of isotherms are observed when the penetrant is a plasticizing or swelling agent for the polymer, like water for the biodegradable polyesters. Table 2 reports the S (10^3^ g/g kPa^−1^) parameters for all the analyzed samples, evaluated according to Equation (2). PHB shows lower sorption than PCL. This is in agreement with the observed lower degree of crystallinity of Poly (3-hydroxybutyrate) (see DSC results), being the crystalline phase impermeable to the penetrant molecules. The blend shows the lowest sorption. We can hypothesize that the interaction between both polymers in the blend, in terms of hydrophilic groups that are shielded or less available to the sorption of water molecules. Following the increasing of sample weight as function of time, it was possible to evaluate the diffusion coefficient, D, at different water pressures. The diffusion coefficient can be evaluated from sorption kinetics, modeled by Fick’s second law solution. From the mass transfer balance, the Fick’s second law can be expressed as Equation (3):

∂C/∂t = D × ∂^2^C/(∂x^2^) (3)

For short times, an approximated form of Equation (3) is the Equation (4):

(4) $$m/m_{\mathrm{eq}}\;=\;(4/d)\;\times \;\sqrt{\left(D\;\times \;t\right)/\pi }$$

The kinetics curves were obtained plotting m/m_eq_ versus $\sqrt{t}$, where m is the water mass absorbed at time t, and m_eq_ is the equilibrium sorbed mass. The evaluation of the diffusion coefficient came from the consideration of the slope (k) of the first part of the reduced weight gain (m/m_eq_) curve versus square root of time by using Equation (5).

D = π × ((k × d)/4)^2^ (5)

**Figure 9:**
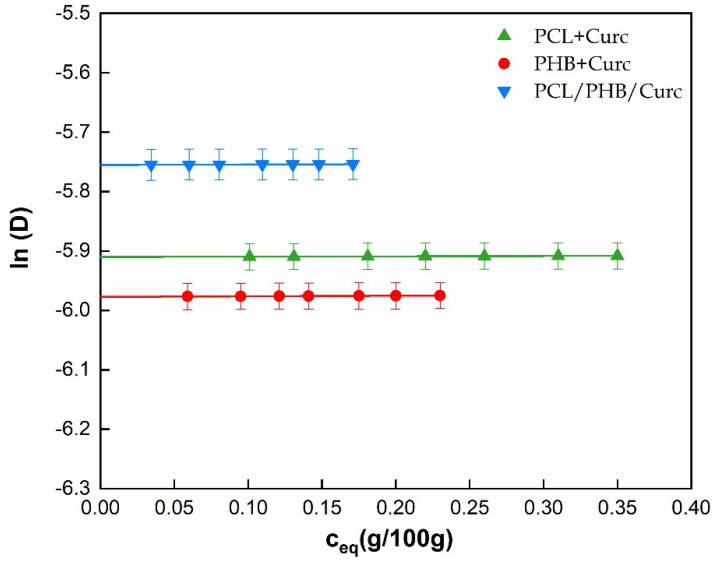
ln (D) as function of equilibrium moisture content Ceq (g/100 g).

Figure 9 reports the ln (D), D in cm^2^/s, as a function of the equilibrium moisture content (Ceq, g/100 g). It is evident that the diffusion is independent of water vapor concentration in the whole investigated water sorbed concentration. We extrapolated the thermodynamic diffusion coefficient, D0, at Ceq = 0. Assuming a good degree of approximation, the D_0_ can be considered equal to D at any vapor pressure. D_0_ values are reported in Table 2. The values of diffusivity are similar for all the samples. The lowest D_0_ is shown by the more crystalline PHB, for the higher tortuous pathway for the travelling water molecules. The blend shows the highest diffusion coefficients. The two polymers being immiscible, during the processing, microvoids can be formed responsible for the increasing in diffusion.

The permeability, product of sorption and diffusion (Equation (6)), was evaluated for all the samples and data reported in Table 2.

P = S × D_0_ (6)

It can be concluded that the variation of permeability in the electrospun membranes is sorption dominated.

**Figure 10:**
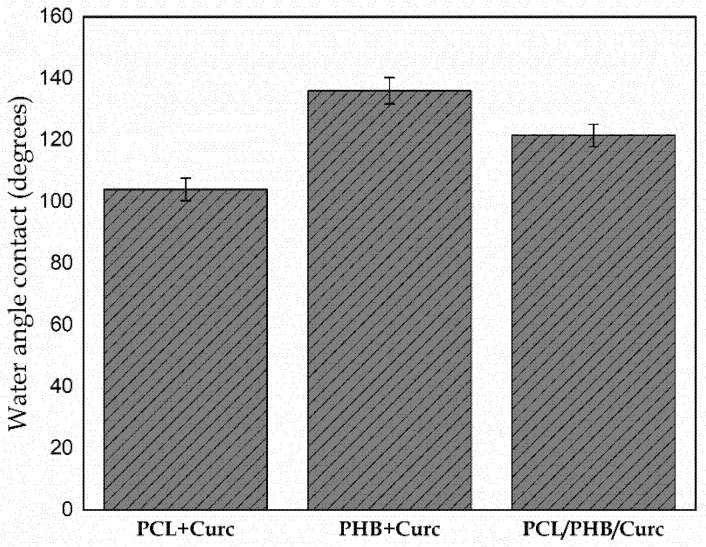
Water contact angle of electrospun membranes.

Figure 10 shows the contact angle of PCL/Curc, PHB/Curc, and the blend PCL/PHB/Curc membranes.

The contact angles of the electrospun PCL membrane is about 104°, in accordance with the scientific literature [57]. The PHB membrane shows a higher hydrophobicity. Its water contact angle (136°) is higher than PCL due to the intrinsic hydrophobicity of its microstructure and the chemical structure of PHB, which allows for an improved wettability. Moreover, the blend PCL/PHB membrane showed, as expected, an intermediate value of water contact angle (121°).

**Figure 11:**
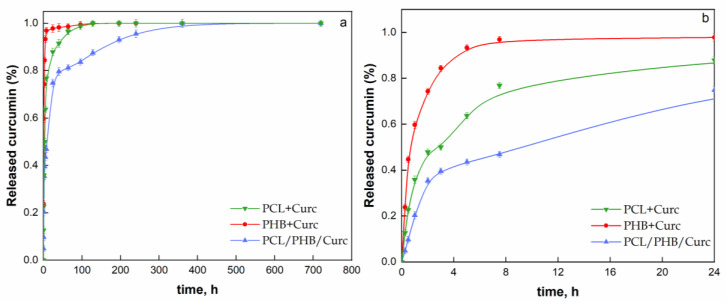
(**a**) Release kinetics of curcumin as function of contact time (h) from the electrospun membranes; (**b**) 24 h chart of curcumin release.

The release kinetics of curcumin from the electrospun membranes were carried out in a pseudo-biologic fluid (PBS/EtOH 70:30 *v*/*v*). The drug release profiles were analyzed and fitted by applying the statistical Weibull model [70,71], expressed by Equation (7) [72]:

m/m_0_ = 1 − exp((−1/A) × (t − T)^b^) (7)

where m is the amount of drug dissolved as a function of time t, m_0_ is total released amount of drug, T parameter represents the latency time resulting from the release process, the scale factor A accounts for the time dependence, and b parameter is related to the drug release mechanism [73]. Moreover, the release phenomenon could be considered the combination of two drug transport phenomena: a diffusion-controlled phase and a relaxation-controlled phase. A modified Weibull model was proposed (Equation (8)) considering that the latency time T is equal to zero and by introducing an additional parameter tm, which considers the time corresponding to maximum cumulative drug release of the stage 1 or the time at which an inflection point in the release curve occurs:

m/m_0_ = θ × (1 − exp((−1/A_1_) × t^b1^) + (1 − θ) × (1 − exp((−1/A_2_) × (t − t_m_)^b2^) (8)

where the first contribution, on the right side, with weight θ represents the diffusion-controlled mechanism while the second one (1 − θ) represents the contribution of the slow release dependent on polymeric chains relaxation. Figure 11a shows the experimental release data of curcumin from the produced electrospun fibrous membranes as function of the release time (h) while Figure 11b shows an expanded 24 h chart of released curcumin.

The fitting of experimental data, by using Equation (8), is reported in Figure 11a as solid line, over the entire time range. The evaluated Weibull model parameters are reported in Table 3.

The release of a compound is supposed to be a gathering of complex phenomena dependent on a series of factors such as physicochemical properties of the solute, structural characteristic of the polymeric system, the release environment and the polymers interaction as well [74]. The parameter θ accounts for the burst release, which occurred at relatively short times. It is worthwhile noting the reduced burst release for PCL/PHB/Curc membrane (44%) compared to PHB + Curc (67%) and PCL + Curc membranes (84%), which allowed for obtaining a more controlled and slow curcumin release. The ratio 1/A_1_ and 1/A_2_ in the Equation (8) could be considered the kinetic constants of the diffusion and relaxation phenomena, respectively. After burst release, the single polymer release kinetics (high 1/A_1_ ratio) allowed for reaching the total released curcumin fraction after about 128 h, for both the systems. For these systems, the relaxation-controlled mechanism (33% for PHB + Curc and 16% for PCL + Curc) is quite negligible since the pretty low release kinetic constants (low 1/A_2_ ratios and low power parameter b). Besides, it is worth noting that the PCL/PHB/Curc membrane shows a slower diffusion release kinetic followed, after a contact time of about 48 h, by a second release step in compliance with the evaluated parameter t_m_. The more controlled release rate of curcumin from the blend system can be due to the formation of weak intermolecular bonds between the two polymeric systems. It follows an increase in mass transfer resistance (low 1/A_1_ value) of curcumin from the bulk of the polymeric network which led to slow its release rate and to reduce the diffusion mechanism contribution (low parameter θ). The second release step contribution (56%) is characterized by a faster relaxation-controlled mechanism (high 1/A_2_ ratio and high power parameter b compared to single polymer systems). Finally, a plateau pattern was reached after about 400 h.

## 4. Conclusions

The paper reported the preparation of electrospun membranes based on PCL, PHB and PCL/PHB blend (50 wt%) loaded with curcumin, as active agent, for biomedical application as drug delivery systems.

- The parameters used to process PHB were significantly different from PCL and the blend PCL/PHB. The temperature used and distance injector–collector were 40 °C and 28 cm, respectively. PCL required lower temperature and distance injector–collector: 25 °C and 18 cm, respectively. The same parameters were used to process the blend PCL/PHB.
- The fibers obtained with PHB showed average diameters ≈ 3.45 µm, the PCL produced fibers of nanometric dimensions ≈ 340 nm, and the PCL/PHB blend also showed average diameter fibers of nanometric dimensions ≈ 520 nm. The same trend was obtained for the fibers’ porosity.
- TGA evaluated on all the samples revealed a slight retard in the degradation of PHB of about 6 °C, due to the better thermal stability of PCL in blend, and a reduction of the degradation temperature of PCL of about 16 °C. This last effect was attributed to the catalytic effect of the degradation products generated by the ester cleavage of PHB due to the β-elimination reaction occurred in the thermal scan in presence of oxygen.
- DSC evaluated on all electrospun materials revealed that in the blend the melting temperatures of both polymers are not significantly influenced by the presence of the second material. The degree of crystallinity for the single polymers in the blend resulted lower than crystallinity of the pure polymers. Each polymer tends to hinder the crystallization of the other one, being immiscible.
- The mechanical properties of the blend resulted intermediate between those of the pure polymers. Elastic modulus (MPa) and stress at break point (MPa), although slightly lower than pure PHB, were much higher than pure PCL. Elongation at break point (mm/mm %) was greatly influenced by the PHB.
- Barrier properties to water vapor (sorption, diffusion and permeability) for the blend resulted improved. The improvement was mainly due to the lowering of sorption. We hypothesized an interaction between both polymers in the blend, in terms of weak bonds between hydrophilic groups, that are shielded or less available to the sorption of water molecules.
- Water contact angle measurements were carried out to estimate the change in wettability of electrospun membranes. The blend PCL/PHB/Curc system showed an intermediate value of water contact angle among the PCL + Curc and PHB + Curc systems.
- The release of curcumin from electrospun membranes was analyzed through the proposed Weibull model. Membranes of pure polymers showed a higher burst release and faster kinetic constants compared to the blend. Besides, for PCL and PHB, the total released curcumin fraction is reached after about 128 h while the system PCL/PHB is characterized by a double step release phenomenon, reaching a plateau after 400 h. Therefore, it was demonstrated that the fabrication of an electrospun blend PCL/PHB allowed to tune the release rate of curcumin for targeted applications.

## Figures and Tables

**Table 1:** Properties and processing parameters of fibrous membranes fabricated by electrospinning.

Sample	Viscosity (mPa·s)	Conductivity (μS/cm)	Temperature (°C)	Relative Humidity (%)	Flow Rate (mL/h)	Distance (cm)	Voltage (kV)
PCL/PHB/Curc	396 ± 18	0.17	25	35	2	18	20/−1
PHB + Curc	465 ± 28	0.26	40	30	3	28	22/0
PCL + Curc	252 ± 15	2.16	25	35	0.5	18	17.5/0

**Table 2:** Sorption, diffusion and permeability coefficients of electrospun mats.

Sample	S (10^3^ g/g kPa^−1^)	D_0_ (cm^2^/s) × 10^6^	P (g/g kPa^−1^) (cm^2^/s) × 10^9^
PCL + Curc	2.10 ± 0.21	1.23 ± 0.42	2.58 ± 0.21
PHB + Curc	1.33 ± 0.26	1.06 ± 0.21	1.41 ± 0.14
PHB/PCL/Curc	1.03 ± 0.19	1.76 ± 0.25	1.81 ± 0.11

**Table 3:** Kinetic parameters evaluated from the fitting process of release data using Equation (8).

Sample	θ	A_1_ (h^b1^)	b_1_	A_2_(h^b2^)	b_2_	t_m_(h)	R^2^
PCL + Curc	0.67	2.30	1.24	18.82	0.005	0	0.998
PHB + Curc	0.84	2.42	0.57	14.10	0.001	0	0.995
PHB/PCL/Curc	0.44	6.25	0.57	9.10	0.25	47.5	0.991
