# Correction: Gorrasi et al. Fabrication and Characterization of Electrospun Membranes Based on “Poly(ε-caprolactone)”, “Poly(3-hydroxybutyrate)” and Their Blend for Tunable Drug Delivery of Curcumin. *Polymers* 2020, *12*, 2239

**DOI:** 10.3390/polym15040939

**Published:** 2023-02-14

**Authors:** Giuliana Gorrasi, Raffaele Longo, Gianluca Viscusi

**Affiliations:** Department of Industrial Engineering, University of Salerno, Via Giovanni Paolo II, 132, 84084 Fisciano, SA, Italy

In the original publication [1], there was a mistake in Figure 2. Figure 2b must be substituted with the following one. The correct legend appears below. The authors state that the scientific conclusions are unaffected. This correction was approved by the Academic Editor. The original publication has also been updated.

## Figures and Tables

**Figure 2:**
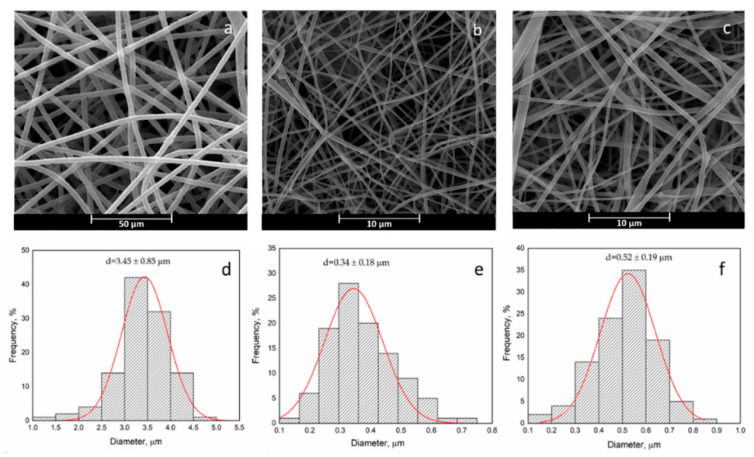
SEM image and fiber diameter distribution: PHB + Curc (**a**,**d**), PCL + Curc (**b**,**e**) and PCL/PHB/Curc (**c**,**f**).
